# Prescribing patterns for treatment of acne vulgaris: A retrospective chart review at an urban public and private hospital

**DOI:** 10.1007/s00403-025-03900-0

**Published:** 2025-02-15

**Authors:** Nicole C. Syder, Arielle Carolina Mora Hurtado, Autumn Saizan, Melissa Gonzalez, Jack Rodman, Nada Elbuluk

**Affiliations:** 1 Department of Dermatology, University of California San Francisco School of Medicine, San Francisco, CA USA; 2 University of Wisconsin School of Medicine and Public Health, Madison, WI USA; 3 Department of Dermatology, Johns Hopkins University School of Medicine, Baltimore, MD USA; 4 Keck School of Medicine, University of Southern California, Los Angeles, CA USA; 5 Southern California Clinical and Translational Science Institute, University of Southern California, Los Angeles, USA; 6 Department of Dermatology, Keck School of Medicine, University of Southern California, 830 S Flower Street, Ste 100, Los Angeles, CA 90017 USA

**Keywords:** Skin of color, Acne, Acne treatments, Acne prescriptions, Postinflammatory hyperpigmentation, Health equity

## Abstract

**Background:**

Studies have found demographic differences in prescribing patterns for certain inflammatory conditions, including acne.

**Objective:**

To investigate acne prescription patterns among patients seen in the private system (PS) and safety-net health care system (SNS) of the University of Southern California (USC).

**Methods:**

This was a multisite, retrospective study of patients obtaining acne care at PS and SNS outpatient dermatology facilities in Los Angeles over a one-year period.

**Results:**

Despite similar acne severity, SNS patients were less often prescribed azelaic acid, benzoyl peroxide/clindamycin, benzoyl peroxide/adapalene, sulfacetamide, topical dapsone, and salicylic acid than PS patients (*p* < 0.001). SNS patients received fewer prescriptions for oral medications including spironolactone, antibiotics, and isotretinoin (*p* < 0.001). Despite similar acne severity, non-White patients were less frequently prescribed topical retinoids (*p* = 0.003), benzoyl peroxide/clindamycin (*p* = 0.003), isotretinoin (*p* < 0.001) and spironolactone (*p* < 0.001) than White patients. Despite higher acne severity among Hispanics/Latinos, they were less often prescribed spironolactone and oral antibiotics than their non-Hispanic/Latino counterparts (*p* = 0.023).

**Conclusions:**

Findings from this study highlight differences in acne prescribing patterns by race/ethnicity and hospital system, which can impact the ability of patients to have successful treatment of their acne and its sequelae.

## Introduction

Acne is a common dermatologic condition with significant importance among skin of color (SOC) patients, with studies reporting acne as a leading dermatological diagnosis in SOC populations [1–3]. In individuals with SOC, acne can be particularly distressing given the potential for the development of sequelae such as post-inflammatory hyperpigmentation (PIH) and keloidal or atrophic scarring [4]. These complications contribute to increased disease burden and can generate negative impacts on quality of life (QOL) [5–7].

Racial and ethnic disparities have been documented in the treatment of inflammatory conditions, including atopic dermatitis, psoriasis, hidradenitis suppurativa, and acne [8–11]. Prior acne studies have reported an underutilization of systemic acne therapeutics by racial and ethnic minorities [11]. In addition, patients with Medicaid insurance have been found to be prescribed certain topical and oral acne therapies at lower rates when compared to their commercially insured counterparts [11]. This study sought to further explore demographic disparities in acne treatment by investigating acne prescription patterns among the private and safety net health care systems of the University of Southern California (USC).

## Methods

### Study design

This was a multisite retrospective chart review investigating acne prescribing patterns among adult patients seen at private and public outpatient dermatology clinics in Los Angeles. Patients meeting eligibility criteria included those ages 18 to 95 years with an acne diagnosis confirmed by ICD diagnoses codes and subcodes. Patients were seen at the USC’s private and safety-net health care systems over a one-year period, from December 2018 to December 2019. This study obtained ethical approval from the USC Institutional Review Board (IRB). The ICD-codes and subcodes, and clinic location were collected for each individual, in addition to demographics of race/ethnicity, age, and gender, as well as preferred language, insurance status, acne severity, over-the-counter treatments (OTC), and prescribed topical and oral acne therapies.

Patients were seen at the Keck Medical Center and Los Angeles (L.A.) County General Medical Center. These are two medical systems served by the same physician population. Keck is a private hospital system (PS) and L.A. General is a public safety net hospital (SNS). The two hospital systems have differences in patient population including those pertaining to insurance status and racial/ethnic backgrounds [12].

### Statistical analysis

Data were summarized using frequency (percent) for categorical variables and mean (SD) for continuous variables. Associations between categorical variables of interest were evaluated using Pearson’s chi-squared or Fisher’s exact test, as appropriate. Data were stratified by race, ethnicity, and medical center type and similar analyses were conducted. All tests were two-sided and a p-value ≤ 0.05 was considered statistically significant (R version 4.2.1).

## Results

### Demographics

A total of 1,355 patients were identified. The average age was 27.0 ± 12.0 years (range:15–78), with two-thirds (66.6%, *n* = 888) being female. The majority of patients self-identified as other race (45.5%, *n* = 532), followed by White (34.7%, *n* = 406), Asian (13.6%, *n* = 159), Black (4.8%, *n* = 56), and more than one race (1.5%, *n* = 17). Ethnically, 20.7% (*n* = 232) of patients identified as Hispanic/Latino (Table 1). Across all patients, the majority had mild or moderate acne (74.8%), and most were being treated with systemic acne therapies (59.1%). Overall, across all patients, procedures were typically not recommended as adjunctive acne therapy with only 7.7% of patients being offered a procedure for their acne (Table 2).

**Table 1:** Acne prescription patterns by race

Variable	N	Patient Race		p-value
		White (*n* = 406)	Non-White (*n* = 764)	
**Acne Severity**				0.598
Improved/Controlled	83	35 (20.0%)	48 (13.9%)	
Mild	252	86 (48.0%)	166 (48.0%)	
Mild to Moderate	41	13 (7.3%)	28 (8.1%)	
Moderate	96	28 (15.6%)	68 (19.7%)	
Moderate to Severe	32	10 (5.6%)	22 (6.4%)	
Severe	21	7 (3.9%)	14 (4.1%)	
**Previous/current over-the-counter medications**				0.020*
None	438	151 (51.4%)	287 (49.8%)	
Benzoyl peroxide	140	35 (11.9%)	105 (18.1%)	
Retinoids	14	8 (2.7%)	6 (1.0%)	
Salicylic acid	50	15 (5.1%)	35 (6.0%)	
OTC acne lines	128	42 (14.3%)	86 (14.9%)	
Glycolic acid	7	3 (1.0%)	4 (0.7%)	
Witch hazel or tea tree oil	8	1 (0.3%)	7 (1.2%)	
Cosmeceuticals	15	10 (3.4%)	5 (0.9%)	
Other/unspecified	73	29 (9.9%)	44 (7.6%)	
**Previous/current prescription medications**				0.022*
None	188	60 (17.1%)	128 (20.2%)	
Accutane	111	37 (10.6%)	74 (11.7%)	
Topical antibiotics	78	23 (6.6%)	55 (8.7%)	
Spironolactone	104	56 (16.0%)	48 (7.6%)	
Oral antibiotics	155	46 (13.1%)	109 (17.2%)	
Retinoids	181	67 (19.1%)	114 (18.0%)	
Benzoyl peroxide/clindamycin combination	46	15 (4.3%)	31 (4.9%)	
Benzoyl peroxide/adapalene combination	28	9 (2.6%)	19 (3.0%)	
Oral contraceptive pills	41	16 (4.6%)	25 (3.9%)	
Topical dapsone	8	4 (1.1%)	4 (0.6%)	
Other/unspecified	45	17 (4.9%)	28 (4.4%)	
**Topical Treatments**				0.003*
None	102	32 (8.4%)	70 (9.6%)	
Retinoids	421	154 (40.2%)	267 (36.5%)	
Benzoyl peroxideBP/clindamycin combination	171	77 (20.1%)	94 (12.8%)	
Azelaic acid gel	26	5 (1.3%)	21 (2.9%)	
Benzoyl peroxide/adapalene combination	63	20 (5.2%)	43 (5.9%)	
Benzoyl peroxide wash	138	41 (10.7%)	97 (13.3%)	
Benzoyl peroxide/erythromycin combination	9	2 (0.5%)	7 (1.0%)	
Sulfacetamide	14	7 (1.8%)	7 (1.0%)	
Ketoconazole wash	0	0	0	
Topical clindamycin	86	25 (6.5%)	61 (8.3%)	
Topical dapsone	14	7 (1.8%)	7 (1.0%)	
Salicylic acid	23	2 (0.5%)	21 (2.9%)	
Other/unspecified	48	11 (2.9%)	37 (5.1%)	
**Systemic Treatments**				< 0.001*
None	385	123 (38.3%)	262 (43.5%)	
Spironolactone	194	92 (28.7%)	102 (16.9%)	
Oral antibiotics	221	66 (20.6%)	155 (25.8%)	
Accutane	92	34 (10.6%)	58 (9.6%)	
Oral contraceptive pills	27	6 (1.9%)	21 (3.5%)	
Other	4	0	4 (0.7%)	
**Previous procedures**				0.379
None	652	222 (90.6%)	430 (88.8%)	
Intralesional steroids	20	5 (2.0%)	15 (3.1%)	
Chemical peels	15	8 (3.3%)	7 (1.5%)	
Lasers	15	5 (2.0%)	10 (2.1%)	
Dermabrasion	3	1 (0.4%)	2 (0.4%)	
Microneedling	8	1 (0.4%)	7 (1.5%)	
Other/unspecified	16	3 (1.2%)	13 (2.7%)	

Numbers represent frequency (column percent)

*Significant at *p* = 0.05 (Fisher’s Exact)

Of note, 165 patients missing race information

**Table 2:** Acne prescription patterns by hospital system

Variable	All Patients (*n* = 1335)	Hospital/Location		P-value
		Public (*n* = 143)	Private (*n* = 1192)	
**Age (years)**	27.0 (12.0)	33.0 (17.8)	27.0 (11.0)	< 0.001*
	(15, 78)	(18, 65)	(15, 78)	
**Gender**				< 0.001*
Female	888 (66.6%)	75 (52.5%)	813 (68.3%)	
Male	444 (33.3%)	67 (46.9%)	377 (31.9%)	
Nonbinary	1 (0.1%)	1 (0.7%)	0	
**Race**				< 0.001*
White	406 (34.7%)	0	406 (39.3%)	
Black/African American	56 (4.8%)	11 (8.1%)	45 (4.4%)	
Asian/Pacific Islander	159 (13.6%)	15 (11.0%)	144 (13.9%)	
More than one	17 (1.5%)	3 (2.2%)	14 (1.4%)	
Other	532 (45.5%)	107 (78.7%)	425 (41.1%)	
**Ethnicity**				< 0.001*
Hispanic/Latino	232 (20.7%)	86 (63.2%)	146 (14.8%)	
Not Hispanic/Latino	890 (79.3%)	50 (36.8%)	840 (85.2%)	
**Preferred Language**				< 0.001*
English	1270 (95.5%)	96 (67.1%)	1174 (98.9%)	
Spanish	51 (3.8%)	43 (30.1%)	8 (0.7%)	
Other	9 (0.7%)	4 (2.8%)	5 (0.4%)	
**Insurance Type**				< 0.001*
None	35 (2.6%)	0	35 (3.0%)	
Medi-Cal	132 (9.9%)	124 (88.6%)	8 (0.7%)	
Medicare	28 (2.1%)	6 (4.3%)	22 (1.9%)	
PPO	622 (46.8%)	0	622 (52.4%)	
EPO	163 (12.3%)	0	163 (13.7%)	
HMO	58 (4.4%)	0	58 (4.9%)	
Self-pay	41 (3.1%)	10 (7.1%)	31 (2.6%)	
Other/Unspecified	249 (18.8%)	0	249 (21.0%)	
**Acne Severity**				0.242
Improved/Controlled	90 (15.2%)	15 (18.5%)	75 (14.6%)	
Mild	286 (48.2%)	37 (45.7%)	249 (48.5%)	
Mild to Moderate	46 (7.7%)	9 (11.1%)	37 (7.2%)	
Moderate	112 (18.9%)	11 (13.6%)	101 (19.7%)	
Moderate to Severe	35 (5.9%)	3 (3.7%)	32 (6.2%)	
Severe	25 (4.2%)	6 (7.4%)	19 (3.7%)	
**Previous/current over-the-counter medications**				< 0.001*
None	491 (49.7%)	85 (60.3%)	406 (47.9%)	
Benzoyl peroxide wash	163 (16.5%)	37 (26.2%)	126 (14.9%)	
Retinoids	19 (1.9%)	0	19 (2.2%)	
Salicylic acid wash	57 (5.8%)	1 (0.7%)	56 (6.6%)	
Over the counter acne lines	142 (14.4%)	15 (10.6%)	127 (15.0%)	
Glycolic acid	7 (0.7%)	0	7 (0.8%)	
Witch hazel or tea tree oil	10 (1.0%)	0	10 (1.2%)	
Cosmeceuticals	18 (1.8%)	0	18 (2.1%)	
Other/unspecified	82 (8.3%)	3 (2.1%)	79 (9.3%)	
**Previous/current prescription medications**				< 0.001*
None	212 (19.0%)	41 (28.7%)	171 (17.6%)	
Oral isotretinoin	126 (11.3%)	9 (6.3%)	117 (12.1%)	
Topical antibiotics	92 (8.3%)	20 (14.0%)	72 (7.4%)	
Spironolactone	115 (10.3%)	3 (2.1%)	112 (11.5%)	
Oral antibiotics	176 (15.8%)	29 (20.3%)	147 (15.1%)	
Retinoids	207 (18.6%)	26 (18.2%)	181 (18.6%)	
Benzoyl peroxide/clindamycin combination	51 (4.6%)	4 (2.8%)	47 (4.8%)	
Benzoyl peroxide/adapalene combination	30 (2.7%)	1 (0.7%)	29 (3.0%)	
Oral contraceptive pills	48 (4.3%)	3 (2.1%)	45 (4.6%)	
Topical dapsone	8 (0.7%)	0	8 (0.8%)	
Other/unspecified	49 (4.4%)	7 (4.9%)	42 (4.3%)	
**Systemic treatment**				< 0.001*
No	426 (40.9%)	94 (66.7%)	332 (36.9%)	
Yes	615 (59.1%)	47 (33.3%)	568 (63.1%)	
**Topical Treatments**				< 0.001*
None	116 (9.2%)	15 (10.5%)	101 (9.0%)	
Retinoids	480 (37.9%)	63 (44.1%)	417 (37.2%)	
Benzoyl peroxide/clindamycin combination	188 (14.9%)	4 (2.8%)	184 (16.4%)	
Azelaic acid gel	32 (2.5%)	0	32 (2.9%)	
Benzoyl peroxide/adapalene combination	76 (6.0%)	1 (0.7%)	75 (6.7%)	
Benzoyl peroxide wash	161 (12.7%)	30 (21.0%)	131 (11.7%)	
Benzoyl peroxide/erythromycin combination	9 (0.7%)	2 (1.4%)	7 (0.6%)	
Sulfacetamide	17 (1.3%)	1 (0.7%)	16 (1.4%)	
Ketoconazole wash	0	0	0	
Topical clindamycin	94 (7.4%)	16 (11.2%)	78 (7.0%)	
Topical dapsone	15 (1.2%)	0	15 (1.3%)	
Salicylic acid	26 (2.1%)	0	26 (2.3%)	
Other/Unspecified	51 (4.0%)	11 (7.7%)	40 (3.6%)	
**Systemic Treatments**				< 0.001*
None	426 (40.9%)	94 (66.7%)	332 (36.9%)	
Spironolactone	216 (20.8%)	11 (7.8%)	205 (22.8%)	
Oral antibiotics	249 (23.9%)	16 (11.4%)	233 (25.9%)	
Oral isotretinoin	112 (10.8%)	12 (8.5%)	100 (11.1%)	
Oral contraceptive pills	32 (3.1%)	7 (5.0%)	25 (2.8%)	
Other	6 (0.6%)	1 (0.7%)	5 (0.6%)	
**Procedure Recommended**				< 0.001*
No	1228 (92.3%)	119 (84.4%)	1109 (93.3%)	
Yes	102 (7.7%)	22 (15.6%)	80 (6.7%)	

Numbers represent median (IQR) (min, max) for continuous variables and frequency (column percent for categorical

*Significant at *p* = 0.05 (Wilcoxon rank sum or Fisher’s exact/Pearson’s χ^2^)

### Private system vs. Safety net system

When stratified by health care system, SNS patients most often self-identified as non-White (100%) and Hispanic/Latino (63.2%), and nearly one-third (30.1%) spoke Spanish as their preferred language (*p* < 0.001). Medi Cal, a Medicaid insurance, was the most popular insurance held by SNS patients (88.6%) compared to those in the PS (0.7%) (*p* < 0.001). In contrast, fewer patients seen in the PS identified as non-White (60.7%) and Hispanic/Latino (14.8%), and few spoke Spanish as their preferred language (0.7%) (*p* < 0.001). PPO insurance was the primary type of insurance held by PS patients (52.4%) compared to those seen in the SNS (0%) (*p* < 0.001). Acne severity did not differ between patients seen in the SNS or PS.

#### Prior treatments

SNS patients (60.3%) were more likely to have no prior use of OTC acne products when compared to PS patients (47.9%) (*p* < 0.001). Of those previously utilizing OTC acne products, OTC acne lines were the most popular amongst PS patients (15.0%), whereas benzoyl peroxide wash was most popular amongst SNS patients (26.2%) (*p* < 0.001).

Regarding prior prescription medications, 28.7% of SNS patients and 17.6% of PS patients had not used any acne prescription in the past (*p* < 0.001). Of those who had used acne prescriptions, oral antibiotics (20.3%) were most frequent amongst SNS patients, whereas retinoids (18.6%) were most common amongst PS patients (*p* < 0.001).

Overall, a history of prior acne procedures was uncommon amongst patients across both health systems. However, SNS patients more frequently had a history of acne procedures than PS patients (14.4% vs. 9.8%, *p* < 0.001). Prior acne procedures reported amongst patients from both health care systems included intralesional triamcinolone, chemical peels, laser, dermabrasion, and microneedling.

#### Topical treatments

Despite having similar acne severity, a higher percentage of SNS patients were not prescribed acne treatments (10.5%) compared to their PS counterparts (9.0%) (*p* < 0.001). More specifically, SNS patients were less often prescribed azelaic acid, benzoyl peroxide/clindamycin combination, benzoyl peroxide/adapalene combination, sulfacetamide, topical dapsone, and salicylic acid than PS patients (*p* < 0.001).

#### Systemic treatments

Patients seen in the SNS were less frequently prescribed systemic therapies for their acne compared to PS patients (33.3% versus 63.1%) (*p* < 0.001). In particular, SNS patients were less commonly prescribed spironolactone, oral antibiotics, isotretinoin, and more often prescribed oral contraceptive pills compared to those seen in PNS (*p* < 0.001). Across both health systems, patients receiving systemic treatments for their acne were more likely to be less than 40 years old (*p* < 0.001), non-Hispanic/Latino (*p* = 0.041), and speak English as their preferred language (*p* < 0.001).

#### Procedural treatments

Interestingly, patients seen in SNS were more often recommended procedural treatment options for their acne than PS patients (15.6% vs. 6.7%, *p* < 0.001).

### Racial and ethnic differences in prescribing patterns

#### Prior treatments

Across both health systems, when stratified by race, patients were not found to differ in acne disease severity. Across both health systems, 51.4% of White patients and 49.8% of non-White patients had no prior history of OTC product use for their acne. Of those who had utilized OTC products in the past, OTC acne lines were the most popular amongst White patients (14.3%), whereas benzoyl peroxide wash was the most popular OTC treatment amongst non-White patients (18.1%) (*p* = 0.02).

Approximately one-fifth of White patients (17.1%) and non-White patients (20.2%) had not used acne prescriptions in the past. Of those who had used acne prescriptions, retinoids were the most frequent topical acne treatment among White and non-White patients (19.1% and 18%, respectively, *p* = 0.022). No significant differences were observed between White and non-White patients having a history of prior procedural therapies for acne.

#### Topical treatments

**Fig. 1:**
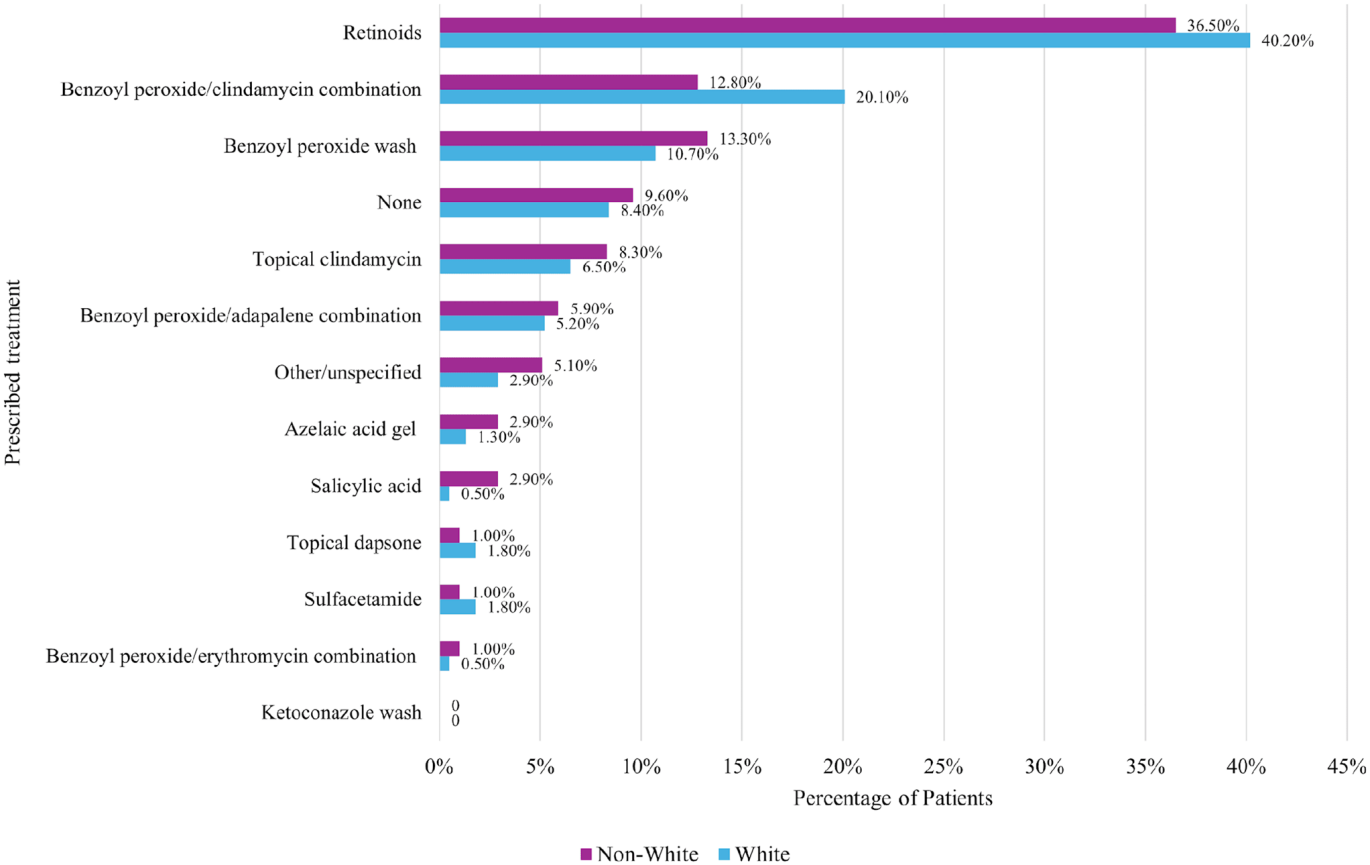
Topical therapies prescribed for white vs. non-white patients

Despite having similar acne severity, non-White patients were less often prescribed topical retinoids than their White counterparts (36.5% vs. 40.2%, respectively, *p* = 0.003). Also, a lower proportion of non-White patients received combination benzoyl peroxide/clindamycin compared to White patients (12.8% vs. 20.1%, respectively, *p* = 0.003) (Fig. 1).

#### Systemic therapies

**Fig. 2:**
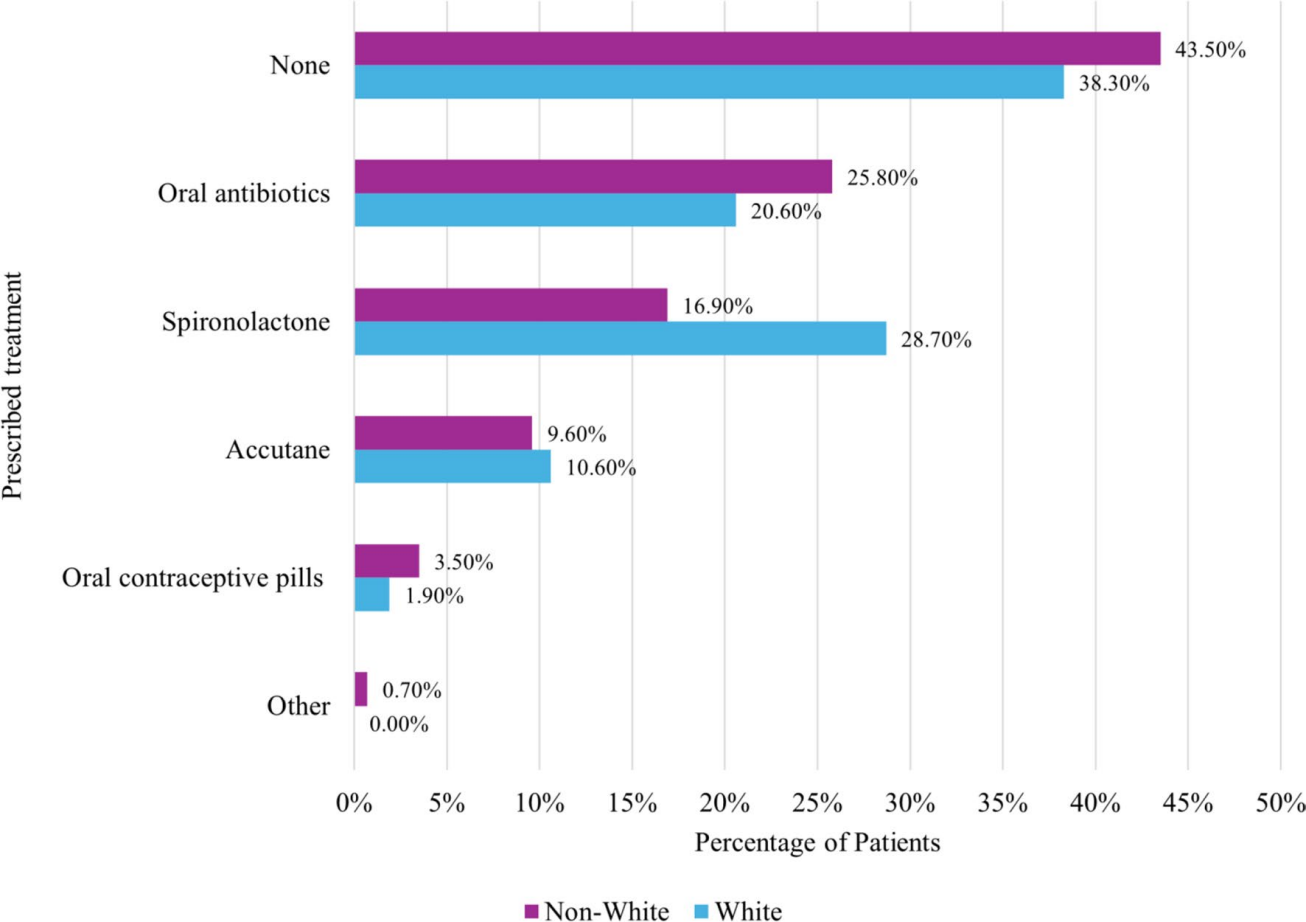
Systemic therapies prescribed for white vs. non-white patients

A high proportion of non-White patients were not prescribed oral acne treatments compared to White patients (43.5% vs. 38.3%, *p* < 0.001). Among patients receiving systemic therapies, non-White patients less frequently received prescriptions for isotretinoin and spironolactone and more often were prescribed oral antibiotics and oral contraceptive pills (*p* < 0.001) (Fig. 2).

Across both health systems, when stratified by ethnicity, Hispanic/Latino patients had more moderate-severe acne compared to non-Hispanic/Latino patients (14.4% vs. 9.4% respectively, *p* = 0.041). Despite this increased disease severity among Hispanics/Latinos, these patients were less often prescribed systemic therapies than their non-Hispanic/Latino counterparts (50.8% vs. 59.1%, respectively, *p* = 0.023). In particular, spironolactone and oral antibiotics were prescribed at lower rates for Hispanics/Latinos (*p* = 0.023).

#### Procedural treatments

Across both health care systems, while procedural therapies were most often recommended for patients seen in SNS, which predominantly serves Hispanic/Latino and Spanish-speaking patients, the patients who most frequently were recommended these procedures were non-Hispanic/Latino (*p* = 0.011) and spoke English as their preferred language (*p* = 0.002).

## Discussion

Despite similar acne severity noted across patients seen in private and public outpatient dermatology clinics, SNS patients were less frequently prescribed certain topical therapies for their acne compared to patients seen in the PS. In addition, SNS patients were also less commonly prescribed oral spironolactone, antibiotics, and isotretinoin compared to PS patients and were more often prescribed oral contraceptive pills. In a retrospective study investigating acne prescription patterns nationally, Medicaid patients with acne were found to be prescribed topical and oral therapeutics at lower rates compared to their commercially insured counterparts [11]. More specifically, Medicaid patients had lower odds of receiving topical retinoids (odds ratio (OR) 0.82), oral antibiotics (OR 0.87), spironolactone (OR 0.50), and isotretinoin (OR 0.43) [11]. Previously, researchers have noted these differences may be related to variations in provider insurance reimbursement [11, 13]. Researchers have suggested that raising Medicaid reimbursement to the level of Medicare rates could help alleviate certain constraints experienced by physicians practicing in under-resourced settings [11, 13–16].

Furthermore, despite similar acne disease severity, our study found that non-White patients less often received certain topical therapies, including combination benzoyl peroxide/clindamycin and topical retinoids than White patients. A study investigating racial and ethnic disparities in prescribed therapeutics for acne, psoriasis, and atopic dermatitis, found that Black patients were less likely to be prescribed adapalene (OR 0.72), tazarotene (OR 0.74), and dapsone (OR 0.39) for their acne compared to White patients [17]. Given that retinoids have efficacy in not only treating comedonal and inflammatory acne but also postinflammatory hyperpigmentation (PIH), a common sequelae of acne in skin of color, not including retinoids in acne regimens for SOC patients not only affects the long-term success of their acne treatment but also PIH, the sequelae of their acne. In addition, given the synergistic effect of benzoyl peroxide and clindamycin in enhancing the efficacy of acne treatment compared to monotherapy with either alone, this also raises concern for undertreatment of acne in patients of color.

Undertreatment of acne in SOC can lead to consequences such as excessive spending on OTC products for self-treatment. A survey study reported that Black respondents had the highest annual spending on OTC products, followed by Asian and White respondents [18]. In addition, the undertreatment of acne in SOC can increase the risk of more severe PIH, which can persist for months to years and generate considerable psychosocial impacts [19]. Previous studies have demonstrated that patients with both acne and PIH tend to have lower quality of life scores than patients with only acne [19].

Moreover, regarding systemic therapies, compared to White patients, non-White patients were less often prescribed isotretinoin and spironolactone and more often prescribed oral antibiotics and oral contraceptive pills for their acne. Similarly, a prior retrospective study found that Black patients with acne were less often prescribed isotretinoin (OR 0.26) than their White counterparts [17]. Another retrospective study revealed that compared to White patients, Black patients had a lower likelihood of being prescribed oral antibiotics (OR 0.80), spironolactone (OR 0.68), combined oral contraceptives (OR 0.64), and isotretinoin (OR 0.39) for their acne [11]. In this same study, racial and ethnic differences in acne prescribing patterns remained unchanged after controlling for mean household income, suggesting these differences are less likely related to financial factors [11]. In regard to ethnicity, despite increased acne severity noted among Hispanics/Latinos in our cohort, these patients were prescribed systemic therapies at lower rates, including spironolactone and oral antibiotics. Similarly, a previous study showed that compared to their White counterparts, Hispanic patients had lower odds of receiving certain systemic acne therapeutics, including combined oral contraceptives (OR 0.62) [11].

Regarding procedural therapies, these therapeutic options were recommended infrequently across both hospital systems. While procedural therapies were more often recommended for patients seen in SNS, which serves a large Hispanic/Latino and Spanish-speaking population, the patients who were more often recommended these procedures were non-Hispanic/Latino and spoke English as their preferred language. Adjunctive therapies such as chemical peels, microneedling, and select laser and light-based therapies can be helpful in the treatment of acne and PIH [7]. Many of these therapies have been found to be safe and efficacious in SOC [7]. To optimize treatment outcomes, patients should be offered these therapy modalities as second-line and adjunctive options as part of a comprehensive treatment approach [7].

Prior studies have identified several factors, including clinician biases, that may contribute to racial/ethnic disparities observed in acne prescription patterns [11, 17, 20–22]. It is well described in the literature that clinician biases can lead to differences in clinical reasoning, diagnostic approaches, and therapeutic recommendations [23, 24]. Further, a distrust in medicine among members of marginalized communities, often stemming from historical medical mistreatment, may exacerbate disparities in acne management [8, 11, 25–27]. In addition, limited patient awareness of available treatment options, especially affecting racial/ethnic minorities and socioeconomically disadvantaged groups, may further exacerbate disparities in acne prescribing practices [8, 11, 17, 27, 28].

Limitations of this study include its single institution and retrospective nature. Further, the racial/ethnic categorization employed may not capture differences in acne prescribing patterns in other racial/ethnic groups not analyzed. Moreover, due to the retrospective nature of the study, acne severity was determined clinically based on the patient’s health record, rather than using objective acne severity scoring methods. Further, our study included data from both new visits and follow-up visits. The inclusion of patients with improved/controlled acne may have potentially led to an underestimation of acne severity. Future studies should consider stratifying results by visit type. In addition, the population of patients visiting USC’s Keck and L.A. General facilities may not be generalizable to the broader Los Angeles population and other populations and settings.

## Conclusion

Limited studies in the U.S. have investigated demographical differences in acne prescription patterns. This study adds to the literature by examining differences in acne prescription patterns by race/ethnicity and hospital system (safety-net vs. private) with consideration of patient acne severity. Findings from this study showed an underutilization of systemic acne treatments, combined anti-inflammatory and topical antibiotic regimens among racial/ethnic minorities and safety-net patients. These findings further highlight a concerning disparity that may contribute to the undertreatment of acne in patients of color and underserved populations. Increased study is needed to understand the factors contributing to disparities in prescribing practices of dermatologists. Advancements in these areas can help promote equitable care of diverse patient populations for this common and burdensome condition which can generate significant impacts on quality of life.

## Data Availability

The data that support the findings of this study are available from the corresponding author upon reasonable request.
